# The associations of shift work exposure and chronotype with sleep problems among Hong Kong nurses: results from the HKNight cohort baseline

**DOI:** 10.1186/s12912-025-03990-1

**Published:** 2025-11-11

**Authors:** Beixi Li, Priscilla Ming Yi Lee, Anke Huss, Yuen Ting Julie Ma, Joey Wing-Yan Chan, Yun Kwok Wing, Lap Ah Tse

**Affiliations:** 1 JC School of Public Health and Primary Care, The Chinese University of Hong Kong, Hong Kong SAR, China; 2 Shenzhen Municipal Key Laboratory for Health Risk Analysis, The Chinese University of Hong Kong, Shenzhen, China; 3 Department of Clinical Medicine, Department of Clinical Epidemiology, Aarhus University, Aarhus, Denmark; 4 Department Population Health Sciences, Utrecht University, Utrecht, Netherlands; 5 Department of Psychiatry, Faculty of Medicine, The Chinese University of Hong Kong, Hong Kong SAR, China; 6 4/F School of Public Health and Primary Care, Prince of Wales Hospital, Sha Tin, N.T., Hong Kong SAR, China

**Keywords:** Work schedule, Night shift work, Chronotype, Sleep quality, Insomnia

## Abstract

**Background:**

Shift work is often associated with poor sleep outcomes; however, findings vary among different individual chronotypes. This study aimed to investigate the associations between various shift work exposures, individual chronotypes, and scale-assessed sleep quality and insomnia among Hong Kong nurses.

**Method:**

The study was conducted between March 2022 and February 2023. The participants completed a self-reported online questionnaire. Sleep outcomes were assessed using the Pittsburgh Sleep Quality Index (PSQI score ≥ 6 indicates poor sleep quality) and the Insomnia Severity Index (ISI score ≥ 8 indicates insomnia). Chronotype was measured using the Munich Chronotype Questionnaire. Multivariate ordinal logistic regression models were used to estimate the associations.

**Results:**

We recruited 718 full-time nurses and 208 daytime office workers. Compared with daytime office workers, night shift nurses had significantly greater odds of having a PSQI score ≥ 6 (adjusted odds ratio [aOR] = 3.38, 95% CI: 2.37–4.81) and an ISI score ≥ 8 (aOR = 1.69, 95% CI: 1.18–2.43). Additionally, workers with an evening chronotype presented greater odds of having a PSQI score ≥ 6 compared with those with a morning type (aOR = 1.69, 95% CI: 1.17–2.44). Among shift nurses, the odds of a PSQI score ≥ 6 increased with the number of night shifts worked in the previous month (*P*_*trend*_ = 0.028). Furthermore, the start time of afternoon shifts demonstrated a quadratic relationship with nurses whose PSQI score was ≥ 6 (*P*_*trend*_ <0.001). Poor mental health significantly mediated the effect of night shift work on poor sleep.

**Conclusion:**

Night shift work and evening chronotype were significantly associated with poor sleep quality and insomnia among Hong Kong nurses, and this association was partially mediated by poor mental health. Optimizing shift schedules (particularly afternoon timing) and enhancing mental health support may mitigate these effects. Chronotype-aligned scheduling may not improve sleep quality among nurses.

**Supplementary Information:**

The online version contains supplementary material available at 10.1186/s12912-025-03990-1.

## Introduction

Poor sleep has emerged as a global public health concern, with 16.6% of adults reporting severe to extreme nocturnal sleep disturbances and 14.5% experiencing clinically significant insomnia, which poses substantial occupational risks among working populations [1, 2]. Shift work, particularly night shift schedules, is strongly associated with sleep disturbances and related health consequences, especially among nurses providing 24/7 patient care [3]. Research indicates that night shift work contributes to sleep deprivation and reduced sleep quality [4], although emerging evidence reveals significant variation in tolerance among workers with different chronotypes [5]. These individual differences influence adaptation to shift work characteristics, including shift frequency (i.e., the number of monthly night shifts) and shift timing (i.e., start and end times) [6].

Chronotypes are typically classified into three main categories: morning, intermediate, and evening types [7]. The evening chronotype, characterized by a later sleep-wake cycle within the 24-hour period, is a risk factor for sleep disturbances in the general population [8, 9]. However, research has shown that among shift workers, those with evening chronotypes experience longer sleep durations and fewer sleep disturbances following night shifts compared with their morning type counterparts, suggesting greater vulnerability to sleep issues among morning type individuals engaged in night shift work [5]. Current evidence further demonstrates that sleep disturbances increase susceptibility to mental health challenges, particularly among both shift workers and individuals with evening chronotypes [8, 9]. These relationships may be compounded by behavioural and environmental factors, including late-evening eating habits, smoking status, napping, and household noise exposure, all of which influence sleep physiology [10–12]. Given their relevance, these factors were accounted for in our analysis. Furthermore, poor mental health is suggested as a potential mediator in the relationship between shift work/evening chronotype and sleep disturbances [10].

A recent intervention study demonstrated that chronotype-aligned shift scheduling (e.g., assigning night shifts to evening type individuals) improves both sleep duration and quality [13]. However, nursing shift schedules differ substantially from those in other industries [14, 15], and critical gaps remain in understanding how monthly night shift frequency, specific shift timing, and shift duration contribute to poor sleep quality and insomnia [6]. This study therefore examines associations between chronotypes and two sleep disturbances, scale-assessed poor sleep quality and insomnia, among Hong Kong nurses, with particular attention given to shift work exposures, including monthly night shift frequency, start and end times of shifts, and shift durations. Our hypothesis is that night shift nurses with an evening chronotype experience significantly more sleep disturbances compared to those with a morning chronotype.

## Materials and methods

### Study participants and data collection

This study presents baseline data from the ongoing prospective cohort, the Hong Kong Nurse Night Shift Cohort (HKNight), which was conducted between March 2022 and February 2023. We recruited local nurses and age-matched (± 2.5 years) daytime office workers with comparable sex distributions and educational attainment. Nurse participants were enrolled through collaboration with the Hong Kong Nurse Association. Daytime office workers (including administrators, research staff, and clerical staff) were recruited by distributing study advertisements using institutional mass mail services to administrative departments of health care institutions, clinics, and public sectors. The eligibility of nurses was based on the following criteria: (1) age ≥ 18 years; (2) current full-time employment; and (3) ≥ 6 months of shift work experience (defined as scheduled work outside 8:00 a.m. to 6:00 p.m.). Daytime workers were required to have no shift work involvement. All eligible participants completed self-administered questionnaires at baseline.

Owing to COVID-19 restrictions in Hong Kong, data collection was conducted electronically via Qualtrics (Provo, Utah, USA). A team of three research assistants with formal training in survey administration provided standardized technical support to the participants. Following a standardized protocol, trained researchers checked the completeness of each submitted Qualtrics-based questionnaire in the same manner and contacted participants if there were any inconsistent or missing data. Adopting this standardized approach to data collection ensured data integrity and reduced information bias. All support staff completed a half-day training protocol covering survey administration ethics and troubleshooting common technical issues.

The comprehensive survey collected data on sociodemographic characteristics, including age, sex, height, weight, education, marital status, monthly income, and self-reported health status. Participants also provided details on their lifetime occupational history, health behaviours such as smoking (defined as consuming at least 20 lifetime packs or smoking weekly for at least one month) and alcohol consumption (defined as drinking at least once a month for six consecutive months), and environmental factors such as perceived noise levels. Mental health status was determined using the Hospital Anxiety and Depression Scale (HADS) to screen for depressive and anxiety symptoms [16]. A score of 8 points or higher on the HADS subscales for anxiety or depression is considered a clinical cut-off point [16]. Poor mental health was defined as having either anxiety or depression.

### Ethics approval and consent to participate

The study adhered to the Declaration of Helsinki and was approved by the Joint CUHK-NTEC CREC (*Ref.*: CREC 2021.228, Joint Chinese University of Hong Kong and New Territories East Cluster Clinical Research Ethics Committee). Signed informed consent was obtained from each study participant after the significance of the study was explained.

### Shift work exposure definition and work timing

In this study, shift work was defined as any work schedule occurring outside the hours of 8:00 a.m. to 6:00 p.m., encompassing three distinct shift types in Hong Kong, including morning shifts (typically starting before 7:00 a.m. and ending at approximately 2:00 p.m.), afternoon shifts (generally beginning at approximately 2:00 p.m. and ending at approximately 9:00 p.m.), and night shifts (usually starting at approximately 9:00 p.m. and ending at 7:00 a.m. the following day). Consistent with International Labour Organization standards, night work was specifically defined as comprising at least three consecutive hours between midnight and 5:00 a.m [17]. Night shift workers in our study population were those engaged in night shifts, either rotating among morning, afternoon, and night shifts or between morning and night shifts. Other shift workers included those rotating between morning and afternoon shifts or working morning or afternoon shifts exclusively.

All participants provided detailed lifetime occupational histories through the baseline questionnaire, including their department of service (i.e., inpatient, outpatient, or emergency room), employment duration, job title, and shift work/night work involvement. For current employment, participants reported daily working hours and weekly work duration (the number of working days and the total working hours). Nurses performing night shifts in their current position provided additional information about their night shift duration. Each nurse submitted a complete 1-month shift schedule for their current position.

Shift timing data were collected using self-reported start and end times for each shift type. We categorized shift timings based on both the observed range of schedules and the distribution of nurses across shifts. Morning shifts were classified by start times (before 7:00 a.m., 7:00 -< 8:00 a.m., 8:00 -< 9:00 a.m., or after 9:00 a.m.) and end times (before 3:00 p.m., 3:00 -< 3:30 p.m., 3:30 -< 4:00 p.m., or after 4:00 p.m.). Afternoon shifts were categorized by start times (before 12 p.m., 12-<1 p.m., or after 2:00 p.m.) and end times (before 9:00 p.m., 9:00-< 9:30 p.m., 9:30-< 10:00 p.m., or after 10:00 p.m.). Night shifts were similarly classified by start times (before 9:00 p.m., 9:00 -< 9:30 p.m., 9:30 -< 10:00 p.m., or after 10:00 p.m.) and end times (before 7:00 a.m., 7:00 -< 7:15 a.m., 7:15-< 7:30 a.m., or after 7:30 a.m.).

### Individual chronotype assessment

The participants completed the validated Chinese version of the Munich Chronotype Questionnaire (MCTQ), which assesses sleep and wake patterns [7, 18]. For daytime office workers, the chronotype was determined using the midpoint between sleep onset and offset on workfree days [7]. Shift nurses’ chronotypes were evaluated using the MCTQ^shift^ [19], which similarly calculates midsleep timing but accounts for variations in sleep patterns following different shift types. This approach recognizes that sleep patterns on free days after afternoon shifts typically show the least disruption from work schedules, providing the most reliable estimate of natural chronotypes in shift-working populations [19].

### Outcomes of interest

The primary outcomes were sleep quality and insomnia severity. Sleep quality was evaluated using the validated Chinese version of the Pittsburgh Sleep Quality Index (PSQI) [20], with total scores ranging from 0 to 21 points. The cut-off value for the PSQI for defining poor sleep quality is 6 points or higher (PSQI score ≥ 6) [20], and scores are further categorized into four ordinal-level categories: good (0–5 points), fairly good (6–10 points), fairly poor (11–15 points), and poor (16–21 points) sleep quality [20].

Insomnia severity was assessed using the Insomnia Severity Index (ISI, with ISI score ≥ 8 as the cut-off value) [21, 22], which classifies symptoms into four categories: no clinically significant insomnia (0–7 points), subthreshold insomnia (8–14 points), moderate clinical insomnia (15–21 points), and severe clinical insomnia (22–28 points) [21, 22]. Owing to the limited number of participants in the severe insomnia category, we combined moderate and severe cases into a single category representing individuals with moderate or severe insomnia symptoms, as per the established ISI categorization [21, 22].

### Statistical analyses

Continuous variables are presented as means ± standard deviations, whereas categorical variables are reported as frequencies and percentages. Baseline characteristics were compared across worker groups (daytime office workers, night shift nurses, and other shift nurses) using one-way ANOVA for continuous variables and chi-square tests for categorical variables.

We analysed the relationships between shift work exposures, chronotypes and sleep disturbances (PSQI score ≥ 6 and ISI score ≥ 8) using ordinal logistic regression, which was selected because our PSQI and ISI scores had naturally ordered categories. Model fit was assessed using likelihood ratio tests and the AIC/BIC, with ordinal regression demonstrating superior fit compared with alternative approaches.

We focused on two key aspects of shift work, core exposure variables and specific timing parameters, while carefully accounting for potential confounding factors, including age, sex, HADS subscale score ≥ 8 (yes, no), household smoking (yes, no), late-evening eating (yes, no), napping behaviours (yes, no), and perceived home noise levels (quiet, relatively quiet, relatively noisy, noisy), which are related to sleep physiology [10–12]. The core exposures are (1) work schedules (daytime office work, night shift work, and other shift work); (2) chronotypes (morning, intermediate, and evening); (3) cumulative years of night work (0 <- 5 years, 5 <- 10 years, and > 10 years); and (4) the number of night shifts in the previous month (1–3 nights, 4–6 nights, and ≥ 7 nights). The specific timing parameters are the start and end times of shift work (as listed in the “Shift work definition and work timing” subsection).

The initial models (Model 1) estimated the associations between sleep outcomes and each core exposure by controlling for only the basic demographic factors (age and sex). Model 2 (fully adjusted model, incorporated a comprehensive set of theoretically relevant covariates selected based on prior evidence): work schedules, chronotype, years of working, and confounding factors. To avoid collinearity and model overfitting, Model 3 (fully adjusted model) included cumulative years of night work, the number of night shifts in the previous month, chronotype, and confounding factors. In the shift timing models, work schedule categories were excluded to separately examine the effects of work start times (Models 4–6) and work end times (Models 7–9). Models 4–6 (fully adjusted models) included morning/afternoon/night shift start time, working hours, the number of shifts in the previous month, chronotype and confounding factors, whereas Models 7–9 (fully adjusted model) included morning/afternoon/night shift off time. A flow diagram of the modelling process is provided in Supplementary Figure S1. Throughout our analyses, we maintained consistent reference categories: daytime office work for work schedule comparisons, morning types for chronotype assessments, and no night shift work for night exposure analyses. We also conducted trend tests to identify potential dose-response relationships between shift work characteristics and sleep outcomes.

We evaluated the potential moderating effects of chronotype using likelihood ratio tests for interaction terms (chronotype × work schedules and chronotype × shift timing), building on evidence that chronotype-aligned scheduling may improve sleep outcomes [13]. We implemented causal mediation analysis using Valeri and VanderWeele’ SAS macros (PROC CAUSALMED) to decompose total effects into natural direct and indirect effects through a counterfactual framework [23]. This approach estimates (1) the natural direct effect of shift work on sleep disturbances (PSQI score ≥ 6 and ISI score ≥ 8) while mental health mediators (HADS-measured depression and anxiety symptoms) are fixed at their unexposed levels, and (2) the natural indirect effect operating through HADS-measured depression and anxiety symptoms [23]. Although originally developed for longitudinal designs, recent methodological work supports its application to cross-sectional data when temporal assumptions are explicitly stated [24]. To ensure that our findings were robust, we repeated the analyses after excluding participants who (1) smoked or had chronic health conditions or family mental health histories, and (2) were nurses working in outpatient or emergency rooms. All the statistical tests used a significance threshold of *p* < 0.05. Analyses were performed using SAS 9.4 (SAS Institute, Inc., Cary, NC), with data visualization conducted via RStudio (v2023.12.1 + 402).

## Results

### Participant characteristics and sleep problems

The present study included 926 participants, comprising 718 shift work nurses and 208 daytime office workers (Supplementary Figure S2). Shift work nurses had a mean age of 33.4 ± 7.5 years, with female night shift nurses comprising 76.2% of all the shift workers. Notably, 80% of the night shift nurses had poor sleep quality (PSQI scores ≥ 6), whereas 56% met the criteria for insomnia (ISI score ≥ 8).

As shown in Table 1, compared with other workers, night shift nurses were significantly younger, exhibited earlier sleep-wake patterns (i.e., morning chronotype), and reported shorter sleep durations. Among shift nurses, 65% had accumulated more than five years of night shift experience. Lifestyle patterns revealed that 39.3% of night shift nurses regularly napped on free days, and 66.7% reported habitual late-evening eating (after 10 p.m.). Night shift nurses experienced higher perceived home noise levels and lower rates of household smoking exposure than daytime office workers.

**Table 1:** Selected characteristics of day workers and shift workers (mean ± sd; n [%])

Categories	Daytimee workers *N* = 208	Night shift nurses *N* = 675	Other shift nurses ^a^ *N* = 43	*p* value
**Mean ± sd**				
Age (Years)	35.6 ± 9.8	33.4 ± 7.6	36.0 ± 7.5	< 0.01
Average sleep duration (Hours)	6.7 ± 1.0	6.5 ± 1.5	7.0 ± 1.2	0.01
PSQI (Points)	6.0 ± 2.5	8.3 ± 3.2	6.3 ± 2.6	< 0.01
ISI (Points)	6.7 ± 5.0	8.8 ± 5.3	6.3 ± 4.1	< 0.01
HADS-Anxiety (Points)	6.4 ± 3.7	7.3 ± 3.7	7.0 ± 4.1	0.01
HADS-Depression (Points)	5.8 ± 3.6	7.4 ± 3.8	6.2 ± 3.8	< 0.01
Years of working (Years)	10.7 ± 9.5	9.6 ± 7.7	11.9 ± 7.3	0.06
Length of a morning shift/day work (hours)	9.4 ± 1.0	8.1 ± 0.7	8.7 ± 0.5	< 0.01
Length of an afternoon shift (hours)	-	8.2 ± 0.7	8.3 ± 0.4	0.60
Length of a night shift (hours)	-	9.9 ± 1.1	-	-
**Frequency (%)**				
Sex (Female)	161 (77.4)	547 (81.0)	39 (90.7)	0.12
Members smoking at home (Yes) ^b^	48 (23.1)	146 (21.6)	5 (11.6)	0.06
Nap on workfree days (Yes)	53 (25.5)	267 (39.3)	15 (34.9)	0.00
Night eating habit (Yes) ^c^	77 (37.0)	428 (63.4)	12 (27.9)	< 0.01
Cumulative night work duration (Years)				
No night work	208 (100.0)	0 (0)	1 (2.3)	< 0.01
0 <- 5 Years	-	242 (35.6)	16 (37.2)	
5 <- 10 Years	-	262 (38.5)	19 (44.2)	
> 10 Years	-	176 (25.9)	7 (16.3)	
Subjective noise level at home				
Quiet	41 (19.7)	68 (10.1)	4 (9.3)	< 0.01
Relatively quiet	123 (59.1)	447 (66.2)	30 (69.8)	
Relatively noisy	38 (18.3)	152 (22.5)	7 (16.3)	
Noisy	6 (2.9)	8 (1.2)	2 (4.7)	
Chronotype				
Morning type	34 (16.4)	243 (36.0)	10 (23.3)	< 0.01
Intermediate type	98 (47.1)	294 (43.6)	25 (58.1)	
Evening type	76 (36.5)	138 (20.4)	8 (18.6)	
Symptoms of depression or anxiety ^d^				
No	136 (65.4)	342 (50.7)	26 (60.5)	< 0.01
Yes	72 (34.6)	333 (49.3)	17 (39.5)	

Abbreviations: sd, standard deviation; PSQI, Pittsburg Sleep Quality Index; ISI, Insomnia Severity Index; HADS, Hospital Anxiety and Depression Scale

^a^ Other shift workers were shift workers who had no nighttime work

^b^ Those who lived in the same household and smoked at home

^c^ Participants who had the habit of eating after 10pm

^d^ Symptoms of depression or anxiety: determined by Hospital Anxiety and Depression Scale. No symptoms of depression or anxiety mean the score of < 8 points; and having symptoms of depression or anxiety means the score of 8 or higher

### Associations between shift work exposures, chronotype, and sleep disturbances

Table 2 presents the adjusted odds ratios (aORs) for associations between shift work characteristics, chronotype, and sleep disturbances. Night shift work demonstrated a significant positive association with poor sleep quality (OR = 3.38, 95% CI: 2.37–4.81) compared with daytime work. Compared with morning chronotype workers, evening types showed greater susceptibility to PSQI scores ≥ 6 (aOR = 1.69, 95% CI: 1.17–2.44). We observed a dose-response relationship between night shift frequency and the odds of poor sleep quality (PSQI scores ≥ 6), with a significant trend of increasing odds across higher frequency categories (*P*_*trend*_ =0.028). Analysis of work timing revealed a significant quadratic relationship between afternoon shift start times and poor sleep quality (*P*_*trend*_ < 0.001), indicating that both early and late afternoon start times were associated with better sleep quality. Cumulative night work duration, morning shift timing, and night shift timing were not significantly associated with poor sleep quality (Supplementary Table S1).

**Table 2:** The association between shift work profiles and chronotype on poor sleep quality (PSQI score ≥ 6) and insomnia (ISI score ≥ 8)

Work schedule/night work exposure	PSQI score ≥ 6			ISI score ≥ 8		
	n1/n2^a^	OR (95% CI) ^b^	OR (95% CI) ^c^	n1/n2^a^	OR (95% CI) ^b^	OR (95% CI) ^c^
**Work schedule** ^**d**^						
Day work	113/95	Ref.	Ref.	77/131	Ref.	Ref.
Night shift work	549/126	3.81 (2.77, 5.22)	**3.38 (2.37**,** 4.81)**	381/294	2.22 (1.62, 3.04)	**1.69 (1.18**,** 2.43)**
Other shift work	24/19	1.02 (0.54, 1.93)	1.05 (0.54, 2.06)	14/29	0.81 (0.40, 1.62)	0.70 (0.33, 1.48)
**Chronotype** ^**d**^						
Morning type	203/84	Ref.	Ref.	156/131	Ref.	Ref.
Intermediate type	313/104	1.14 (0.85, 1.52)	1.34 (0.98, 1.83)	200/217	0.75 (0.56, 1.00)	**0.71 (0.52**,** 0.97)**
Evening type	170/52	1.24 (0.88, 1.75)	**1.69 (1.17**,** 2.44)**	116/106	0.85 (0.61, 1.20)	0.92 (0.64, 1.33)
**Number of night work last month** ^**e**^						
No night work	137/114	Ref.	Ref.	91/160	Ref.	Ref.
1–3 nights	136/36	3.08 (2.09, 4.55)	**2.41 (1.19**,** 4.89)**	95/77	2.15 (1.46, 3.16)	1.82 (0.85, 3.93)
4–6 nights	374/86	3.97 (2.89, 5.44)	**3.57 (1.84**,** 6.94)**	263/197	2.33 (1.70, 3.18)	**2.36 (1.15**,** 4.88)**
≥ 7 nights	39/4	5.44 (2.95, 10.03)	**5.23 (2.18**,** 12.56)**	23/20	2.59 (1.43, 4.70)	**2.68 (1.07**,** 6.69)**
p for trend		0.083	**0.028**		0.555	0.280
**Work start time: Afternoon shift** ^**f**^						
<- 12 p.m.	6/6	0.20 (0.06, 0.64)	**0.14 (0.04**,** 0.49)**	2/10	0.16 (0.03, 0.75)	**0.11 (0.02**,** 0.61)**
12 p.m. <- 1 p.m.	203/47	Ref.	Ref.	140/110	Ref.	Ref.
1 p.m. <- 2 p.m.	257/54	1.16 (0.84, 1.60)	1.53 (0.97, 2.41)	178/133	1.08 (0.79, 1.48)	1.25 (0.79, 2.00)
> 2 p.m.	84/26	0.88 (0.56, 1.36)	1.00 (0.59, 1.72)	62/48	1.04 (0.68, 1.60)	1.04 (0.60, 1.80)
p for trend		0.002	**< 0.001**		0.019	**0.005**
**Off-work time: Afternoon shift** ^**g**^						
<- 9 p.m.	44/22	Ref.	Ref.	31/35	Ref.	Ref.
9 p.m. <- 9.30 p.m.	169/35	2.10 (1.22, 3.63)	**2.32 (1.31**,** 4.11)**	113/91	1.35 (0.79, 2.31)	1.68 (0.93, 3.00)
9.30 p.m. <- 10 p.m.	228/51	2.22 (1.31, 3.77)	**2.39 (1.29**,** 4.44)**	159/120	1.46 (0.87, 2.46)	1.79 (0.94, 3.38)
> 10 p.m.	109/25	2.25 (1.26, 4.02)	**2.28 (1.21**,** 4.29)**	79/55	1.58 (0.89, 2.78)	1.81 (0.95, 3.48)
p for trend		0.749	0.937		0.454	0.743

Abbreviation: OR, odds ratio; CI, confidence interval; Ref., reference; PSQI, Pittsburg Sleep Quality Index; ISI, Insomnia Severity Index

^a^ n1/n2: Number of cases over number of non-cases

^b^ Model 1: adjusting for age (18 <- 30, 30 <- 40, 40 <- 50, > 50) and sex

^c^ Odds ratio and 95% CI of the multivariate adjusted models

^d^ Model 2: additionally adjusted for work schedule (daytime work, night shift work, other shift work), chronotype (morning type, intermediate type, evening type), Hospital anxiety and Depression Scale subscale score ≥ 8 (yes, no), whether people living in the same household smoked (yes or no), the habit of eating after 10 pm (yes or no), napping habit on workfree days (yes or no), years of working (0 <- 1 year, 1 <- 5 years, 5 <- 10 years, > 10 years), noise level at home (quiet, relatively quiet, relatively noisy, noisy)

^e^ Model 3: Remove work schedule and years of working in Model 2 and add cumulative night work duration and the number of night shift last month

^f^ Model 5: Remove work schedule and years of working in Model 2 and add afternoon shift start time, afternoon shift duration, the number of afternoon shift last month

^g^ Model 8: Remove work schedule and years of working in Model 2 and add afternoon shift off time, afternoon shift duration, the number of afternoon shift last month

The associations among shift work characteristics, chronotype, and insomnia followed similar patterns to those observed for poor sleep quality, but with attenuated effect sizes (Table 2). Notably, intermediate chronotype workers had lower odds of having insomnia (ISI score ≥ 8) compared with morning types (aOR = 0.71, 95% CI: 0.52–0.97). Supplementary Table S1 presents findings on the association between cumulative night work duration, morning and shift timings, and insomnia (ISI score ≥ 8).

### Mediation effects of mental health on sleep disturbances

Poor mental health significantly mediated the relationship between shift work and sleep disturbances, with depression explaining 23.7% for poor sleep quality (PSQI score ≥ 6) and 70.7% of the risk for insomnia (ISI score ≥ 8) among night shift workers (Table 3). Anxiety showed weaker but still significant mediation effects (15.8% for PSQI scores ≥ 6, 51.1% for ISI scores ≥ 8). Poor mental health did not demonstrate statistically significant mediation effects on the associations between cumulative night work duration, shift timings, and sleep disturbances (Supplementary Table S2).

**Table 3:** Estimation of mediation effects of poor mental health on poor sleep quality (PSQI score ≥ 6) and insomnia (ISI score ≥ 8)

Exposure	Mediator	PSQI score ≥ 6		ISI score ≥ 8	
		Percentage Mediated	p value	Percentage Mediated	p value
Work schedule ^a^	Depression	**23.7 (6.6)**	**< 0.001**	**70.7 (26.3)**	**0.007**
	Anxiety	**15.8 (6.8)**	**0.020**	**51.1 (22.3)**	**0.022**
Number of night shift last month ^b^	Depression	**24.3 (6.4)**	**< 0.001**	**48.4 (14.0)**	**0.001**
	Anxiety	9.9 (6.6)	0.135	20.8 (12.9)	0.108

Abbreviation: PSQI, Pittsburg Sleep Quality Index; ISI, Insomnia Severity Index

^a^ Model 2: Model adjusted for age, sex, work schedule, chronotype, whether people living in the same household smoked, the habit of eating after 10 pm, napping habit during workfree days, years of working, noise level at home

^b^ Model 3: Remove work schedule and years of working in Model 2 and add cumulative night work duration and the number of night shift last month

### Differential effects of shift work on sleep by chronotype and sensitivity analyses

**Fig. 1:**
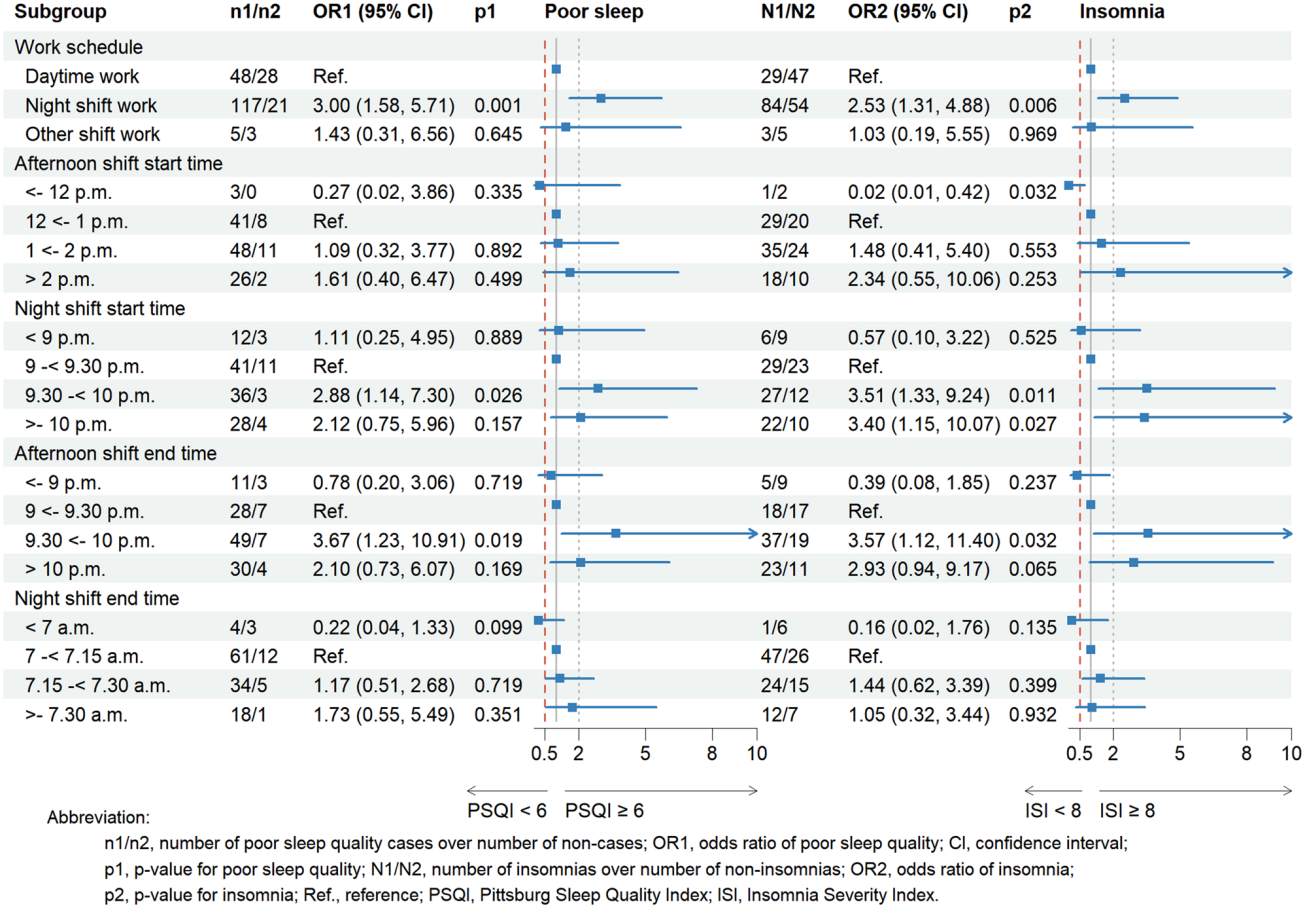
The association between work schedules and shift timing of afternoon shift and poor sleep quality (PSQI score ≥ 6) and insomnia (ISI score ≥ 8) stratified by evening chronotype. Notes:. **a**. Work schedule model: adjusted for age, sex, cumulative years of night work, number of night shifts in the previous month, chronotype, hospital anxiety and depression scale subscale score, whether people living in the same household smoked, the habit of eating after 10 pm, napping habit on workfree days, noise level at home. **b**. Afternoon shift start time model: adjusted for age, sex, afternoon shift start time, afternoon shift working hours, number of afternoon shifts in the previous month, chronotype, Hospital anxiety and depression scale subscale score, whether people living in the same household smoked, the habit of eating after 10 pm, napping habit on workfree days, noise level at home. **c**. Night shift start time model: adjusted for age, sex, night shift start time, night shift working hours, number of night shifts in the previous month, chronotype, Hospital anxiety and depression scale subscale score, whether people living in the same household smoked, the habit of eating after 10 pm, napping habit on workfree days, noise level at home. **d**. Afternoon shift end time model: adjusted for age, sex, afternoon shift end time, afternoon shift working hours, number of afternoon shifts in the previous month, chronotype, Hospital anxiety and depression scale subscale score, whether people living in the same household smoked, the habit of eating after 10 pm, napping habit on workfree days, noise level at home. **e**. Night shift end time model: adjusted for age, sex, night shift end time, night shift working hours, number of night shifts in the previous month, chronotype, Hospital anxiety and depression scale subscale score, whether people living in the same household smoked, the habit of eating after 10 pm, napping habit on workfree days, noise level at home

Supplementary Table S3 shows the associations between shift work characteristics and sleep outcomes stratified by chronotype. Among evening chronotype workers, we observed consistent patterns of associations for both poor sleep quality (PSQI score ≥ 6) and insomnia (ISI score ≥ 8) across afternoon and night shift schedules (Fig. 1). Sensitivity analyses confirmed the robustness of these findings, demonstrating stable associations between shift work exposures and sleep disturbances (Supplementary Table S4-S5).

## Discussion

This study examined the relationships between chronotype and sleep disturbances among Hong Kong nurses across different shift work exposure profiles. Our results demonstrated that both night shift work and evening chronotype were independently associated with poorer sleep quality. We identified a dose-response relationship, with sleep quality deteriorating progressively as the monthly night shift frequency increased. The timing of afternoon shifts revealed that earlier start times appeared protective for sleep quality (although not insomnia severity). Mediation analysis revealed that poor mental health partially explains the pathway between night shift exposure and sleep disturbances.

The PSQI and ISI, two widely used sleep assessment scales, capture related but distinct dimensions of sleep pathology, reflecting the conceptual distinction between poor sleep quality and insomnia [22, 25]. The PSQI serves as a broader composite measure, evaluating overall sleep quality and habitual disturbances across seven components, including latency, duration, efficiency, and daytime dysfunction [20, 25]. In contrast, the ISI functions as a more specific instrument, primarily assessing the severity of core insomnia symptoms (difficulty initiating and maintaining sleep, early awakening) and their subjective impact on daytime functioning and distress [22]. Consequently, although the two scales are significantly correlated (*r* = 0.68) and a high PSQI score can indicate insomnia [26], it can also reflect other causes of poor sleep (e.g., sleep deprivation due to schedule or environmental factors) that do not meet the specific symptom-based and distress/impairment thresholds central to the definition of insomnia syndrome [27].

### Prevalence of sleep disturbances among Hong Kong nurses

Our study revealed substantially elevated rates of sleep disturbances among Hong Kong nurses, with 80% exhibiting poor sleep quality (PSQI score ≥ 6) and 56% meeting clinical insomnia criteria (ISI score ≥ 8), both of which are consistently above international prevalence ranges (36–72% and 9–50%, respectively) [2, 14, 28]. These findings likely reflect Hong Kong’s demanding healthcare environment, where nurses typically manage 8–12 patient loads alongside approximately 15% staff shortages [29]. The elevated rates on both scales in our nursing sample suggest a population experiencing both a generalized deficit in restorative sleep and a high burden of clinical-grade insomnia symptoms. The marked contrast with our daytime administrative controls (including administrative staff in healthcare authorities, research staff in educational institutions, etc.), who demonstrated relatively better sleep profiles concurrent with their standardized 9 a.m.-6 p.m. schedules and lower stress levels, highlights the differential working conditions associated with shift work within this high-pressure context.

Similar to other studies conducted during the COVID-19 pandemic, subject recruitment and response rates may pose a concern [30]. We utilized an online questionnaire to minimize the impact of the pandemic, and this approach resulted in a response rate comparable to that of the non-pandemic period [31]. In addition to nurses, the pandemic may also introduce additional stress for office workers, potentially creating a nondifferential bias between our nursing and office worker samples, which could ultimately lead the effect estimate toward null [32].

### Chronotype variations and sleep disturbances

Our findings regarding sleep quality align with existing evidence demonstrating that night shift work and evening chronotype disrupt circadian rhythms, leading to poor sleep quality [15, 33]. However, the relationship between chronotype and insomnia symptoms within the shift work population remains understudied. While Chan et al. reported greater insomnia severity in evening-types [34], their study was conducted in a clinical sample of insomnia patients, which limits direct comparability to our occupational cohort. In our sample of Hong Kong nurses, we observed no significant association between evening chronotype and insomnia. This may suggest that, in a population already exposed to severe circadian misalignment, the added vulnerability associated with evening chronotype may be less pronounced. Conversely, we observed that intermediate chronotypes presented a reduced risk of insomnia, potentially reflecting greater sleep-wake flexibility in this group [10].

### Night shift frequency and sleep disturbances

The frequency of night shifts emerged as a significant risk factor for sleep disturbances, with a clear dose-response relationship observed for poor sleep quality (PSQI score ≥ 6), which deteriorated progressively with increasing night shift frequency. In contrast, no clear linear trend was observed for insomnia and frequent night shifts, suggesting that the risk for insomnia may reach a threshold or be influenced by factors beyond shift frequency, such as individual vulnerability or recovery opportunities [35]. This distinction may help explain inconsistent findings in literature [6]. For instance, while Garde et al. reported no changes in sleep quality with consecutive night shifts among police officers, a finding potentially explained by the controlled and predictable nature of their work schedules [36], a larger-scale study of Korean hospital nurses (*n* = 33 669) reported a significant association with insomnia [37]. This discrepancy underscores that the impact of shift frequency is not uniform but may be critically moderated by occupational context, including physical and cognitive demands of the job and the predictability of the roster [6, 36].

### Shift timing and sleep disturbances

Our findings also contribute to the predominant research focus on shift work by identifying afternoon shift timing as a significant yet under-investigated risk factor for sleep disturbances [38]. Specifically, we found that afternoon shifts starting before 12 p.m. were associated with the lowest risk of poor sleep quality (PSQI score ≥ 6) and insomnia (ISI score ≥ 8). Although the small sample size in the early afternoon group necessitates validation in larger cohorts, it is plausible that the delayed completion time of later afternoon shifts may contribute to sleep disturbances through both social and physiological pathways. Socially, this atypical schedule creates misalignment with societal norms, leading to social isolation and conflict with family activities, which are known stressors for pre-sleep cognitive arousal [39]. Physiologically, these shifts can truncate the window for wind-down routines and push sleep onset into the late evening or early night, conflicting with the natural drop in core body temperature that facilitates sleep initiation [35]. The combination of this physiological misalignment and social stress likely exacerbates sleep challenges in this group.

### Poor mental health as a mediator of sleep disturbances

Our mediation analysis revealed that poor mental health mediated approximately 70% of the risk for insomnia (ISI score ≥ 8), compared to 24% for poor sleep quality (PSQI score ≥ 6). This pronounced difference suggests that insomnia may arise more from psychological distress (e.g., anxiety and depression) [34, 40], whereas general sleep quality is more directly affected by circadian disruption [5]. Additionally, the mediation was particularly stronger for work schedule effects than for night shift frequency, indicating that mental health plays a greater role in the impact of chronic shift work than in acute exposure [10, 41]. Collectively, these findings highlight the importance of addressing both scheduling factors and mental health support to improve various sleep outcomes in this nursing populations [10, 40].

### Potential relevance of shift work disorder and sleep disturbances

The high prevalence of poor sleep quality and insomnia symptoms observed in our sample prompts consideration of shift work disorder (SWD), a circadian rhythm sleep-wake disorder defined by insomnia and/or excessive sleepiness resulting from a misalignment between the work schedule and the body’s circadian clock [42, 43]. Research indicates that shift workers, particularly those on night shifts, are at a high risk for SWD, which is characterized by excessive sleepiness and insomnia [44, 45]. While our methodological design does not allow for a formal diagnosis of SWD, the profound sleep disturbances we document are a hallmark of this condition. It is plausible that a substantial proportion of the sleep problems reported by our participants may be attributable to the underlying circadian disruption central to SWD, highlighting the extreme challenge that rotating shift work poses to sleep health in this nursing population.

### Strengths and limitations

This study has several strengths. First, we identified afternoon shift timing as a significant factor associated with poor sleep quality, providing actionable insights for optimizing shift schedules to improve nurse adaptation. Second, our sample size represents one of the most comprehensive studies of shift work nurses and daytime workers in Hong Kong, exceeding previous local investigations [32, 46]. Third, our exposure assessment incorporated detailed 1-month shift schedules, enabling precise quantification of night work intensity while minimizing misclassification bias. The inclusion of both inpatient (88%) and outpatient/operation room nurses (12%) captures diverse clinical stressors, from chronic ward overload to emergency department unpredictability, suggesting that findings apply across hospital environments.

Several limitations warrant consideration. The cross-sectional design limits causal inferences. However, as this study provides baseline cohort data, follow-up studies will validate these findings. It is important to note that while the ISI is a validated screening tool for insomnia, a score in the “moderate” or “severe” range indicates clinically significant insomnia symptoms and not a formal clinical diagnosis of insomnia disorder, which would require a more comprehensive assessment including strict criteria for daytime functional impairment and duration. Our design cannot fully distinguish between these possibilities, although the ISI remains a meaningful measure for sleep disturbances in occupational context. Our study may have been underpowered to detect subtle sex-specific effects given the smaller male subgroup. However, the robust consistency of our primary findings across analytical approaches supports their validity. Future research with balanced sex representation could help clarify whether the trends we observed represent true null effects or have limited statistical power. Our study lacked occupation-specific stress measures. Although our sensitivity analysis revealed consistent results across clinical settings, we noted that emergency room nurses are more likely to face different stressors than general ward nurses are. The inclusion of HADS scores in our models helps account for underlying mental health components that often correlate with work-related stress [47]. We did not collect specific data on caffeine consumption, although our behavioural and dietary assessment captured other relevant information, such as smoking, alcohol consumption, and late-evening eating habits, that may influence sleep. Our shift timing analysis did not account for overtime work, which may attenuate the observed associations towards the null. Finally, while our findings capture sleep disturbances linked to shift work, the absence of formal SWD diagnostics (e.g., ICSD-3 or DSM-5) limits direct clinical generalizability. Despite this, our use of the well-validated PSQI and ISI provides a robust measure of sleep quality and insomnia symptoms fundamental to shift work-related pathology [20]. Future research would benefit from incorporating SWD-specific diagnostic criteria to further clarify the clinical implications for shift work nurses.

## Conclusion

Our findings demonstrated that night shift work and evening chronotype were significantly associated with poor sleep quality and insomnia among Hong Kong nurses, whereas these associations were partially mediated by poor mental health. These results suggest two possible intervention targets for future studies: optimizing shift scheduling (possibly adjusting for afternoon shift timing) to better align with psychosocial factors and implementing mental health support programs. Evidence-based scheduling practices that consider both individual and operational demands could help mitigate sleep disturbances while maintaining health care services.

## Supplementary Information

Below is the link to the electronic supplementary material.

Supplementary Material 1

## Data Availability

The data and materials used in this study are available upon request.
