# Supplementary material for: The associations of shift work exposure and chronotype with sleep problems among Hong Kong nurses: results from the HKNight cohort baseline
Source: BMC Nurs. 2025 Nov 11;24:1386. doi: 10.1186/s12912-025-03990-1 (PMC12606913; doi:10.1186/s12912-025-03990-1)

*Supplementary Table S1* The main effects of work start time and work-off time on poor sleep quality and insomnia.

| **Time** | **PSQI score ≥ 6** | | | **ISI score ≥ 8** | | |
| --- | --- | --- | --- | --- | --- | --- |
|  | **n1/n2^a^** | **OR (95% CI) ^b^** | **OR (95% CI)** | **n1/n2^a^** | **OR (95% CI) ^b^** | **OR (95% CI)** |
| **Cumulative night work duration ^c^** | | | | | | |
| **No night work** | 114/95 | Ref. | Ref. | 77/132 | Ref. | Ref. |
| **0 <- 5 Years** | 208/50 | 2.82 (1.90, 4.20) | 0.92 (0.45, 1.86) | 148/110 | 2.00 (1.34, 2.97) | 0.76 (0.35, 1.66) |
| **5 <- 10 Years** | 220/61 | 3.61 (2.49, 5.24) | 1.18 (0.58, 2.39) | 145/136 | 1.87 (1.29, 2.71) | 0.76 (0.35, 1.66) |
| **> 10 Years** | 144/34 | 4.10 (2.69, 6.26) | 1.13 (0.52, 2.46) | 102/76 | 2.66 (1.75, 4.06) | 0.96 (0.41, 2.23) |
| **p for trend** |  | 0.127 | 0.409 |  | 0.234 | 0.385 |
| **Work start time** | | | | | | |
| **Morning shift/day work ^d^** | | | | | | |
| **< 7 a.m.** | 56/15 | 2.51 (1.39, 4.52) | 1.83 (0.74, 4.50) | 36/35 | 1.64 (0.91, 2.96) | 1.10 (0.43, 2.80) |
| **7 -< 8 a.m.** | 486/115 | 3.53 (2.34, 5.32) | **2.21 (1.04, 4.73)** | 343/258 | 2.22 (1.46, 3.36) | 1.46 (0.65, 3.25) |
| **8 -< 9 a.m.** | 83/63 | 0.97 (0.60, 1.58) | 1.18 (0.70, 1.98) | 52/94 | 0.96 (0.58, 1.60) | 1.06 (0.60, 1.86) |
| **>- 9 a.m.** | 61/46 | Ref. | Ref. | 41/66 | Ref. | Ref. |
| **p for trend** |  | <0.001 | 0.065 |  | 0.001 | 0.188 |
| **Night shift ^e^** | | | | | | |
| **< 9 p.m.** | 44/14 | Ref. | Ref. | 26/32 | Ref. | Ref. |
| **9 -< 9.30 p.m.** | 238/57 | 1.21 (0.69, 2.10) | 1.24 (0.67, 2.31) | 161/134 | 1.43 (0.82, 2.48) | 1.58 (0.83, 3.00) |
| **9.30 -< 10 p.m.** | 166/28 | 1.80 (1.01, 3.21) | 1.61 (0.83, 3.13) | 124/70 | 2.03 (1.15, 3.59) | **2.03 (1.02, 4.03)** |
| **>- 10 p.m.** | 101/27 | 1.23 (0.67, 2.26) | 1.20 (0.56, 2.56) | 70/58 | 1.59 (0.87, 2.89) | 1.56 (0.71, 3.43) |
| **p for trend** |  | 0.024 | 0.127 |  | 0.075 | 0.159 |
| **Off-work time** | | | | | | |
| **Morning shift/day work ^f^** | | | | | | |
| **< 3 p.m.** | 180/44 | Ref. | Ref. | 124/100 | Ref. | Ref. |
| **3 -< 3.30 p.m.** | 119/33 | 0.95 (0.64, 1.43) | 0.86 (0.54, 1.35) | 84/68 | 0.99 (0.67, 1.47) | 0.95 (0.60, 1.51) |
| **3.30 -< 4 p.m.** | 213/47 | 0.99 (0.70, 1.41) | 0.86 (0.51, 1.44) | 148/112 | 0.99 (0.70, 1.39) | 0.94 (0.55, 1.59) |
| **>- 4 p.m.** | 174/115 | 0.36 (0.25, 0.50) | 0.78 (0.38, 1.60) | 116/173 | 0.54 (0.38, 0.76) | 1.00 (0.48, 2.08) |
| **p for trend** |  | <0.001 | 0.768 |  | 0.002 | 0.852 |
| **Night shift ^g^** | | | | | | |
| **< 7 a.m.** | 25/9 | Ref. | Ref. | 15/19 | Ref. | Ref. |
| **7 -< 7.15 a.m.** | 290/70 | 1.53 (0.77, 3.04) | 1.51 (0.74, 3.09) | 208/152 | 1.54 (0.78, 3.05) | 1.93 (0.92, 4.05) |
| **7.15 -< 7.30 a.m.** | 153/35 | 1.89 (0.93, 3.87) | 1.50 (0.71, 3.16) | 105/83 | 1.50 (0.74, 3.04) | 1.58 (0.73, 3.42) |
| **>- 7.30 a.m.** | 81/12 | 1.67 (0.77, 3.60) | 1.47 (0.66, 3.29) | 53/40 | 1.48 (0.69, 3.18) | 1.73 (0.75, 3.97) |
| **p for trend** |  | 0.345 | 0.910 |  | 0.870 | 0.459 |

Abbreviation: OR, odds ratio; CI, confidence interval; Ref., reference; PSQI, Pittsburg Sleep Quality Index; ISI, Insomnia Severity Index.

^a^ n1/n2: Number of cases over number of non-cases.

^b^ Model 1: adjusting for age and sex.

^c^ Model 3: adjusted for age, sex, cumulative years of night work, number of night shifts in the previous month, chronotype, Hospital anxiety and Depression Scale subscale score ≥ 8 (yes, no), whether people living in the same household smoked (yes or no), the habit of eating after 10 pm (yes or no), napping habit on workfree days (yes or no), noise level at home (quiet, relatively quiet, relatively noisy, noisy).

^d^ Model 4: adjusted for age, sex, morning shift start time, morning shift working hours, number of morning shifts in the previous month, chronotype, Hospital anxiety and Depression Scale subscale score ≥ 8 (yes, no), whether people living in the same household smoked (yes or no), the habit of eating after 10 pm (yes or no), napping habit on workfree days (yes or no), noise level at home (quiet, relatively quiet, relatively noisy, noisy).

^e^ Model 6: adjusted for age, sex, night shift start time, night shift working hours, number of night shifts in the previous month, chronotype, Hospital anxiety and Depression Scale subscale score ≥ 8 (yes, no), whether people living in the same household smoked (yes or no), the habit of eating after 10 pm (yes or no), napping habit on workfree days (yes or no), noise level at home (quiet, relatively quiet, relatively noisy, noisy).

^f^ Model 7: adjusted for age, sex, morning shift end time, morning shift working hours, number of morning shifts in the previous month, chronotype, Hospital anxiety and Depression Scale subscale score ≥ 8 (yes, no), whether people living in the same household smoked (yes or no), the habit of eating after 10 pm (yes or no), napping habit on workfree days (yes or no), noise level at home (quiet, relatively quiet, relatively noisy, noisy).

^g^ Model 9: adjusted for age, sex, night shift end time, night shift working hours, number of night shifts in the previous month, chronotype, Hospital anxiety and Depression Scale subscale score ≥ 8 (yes, no), whether people living in the same household smoked (yes or no), the habit of eating after 10 pm (yes or no), napping habit on workfree days (yes or no), noise level at home (quiet, relatively quiet, relatively noisy, noisy).

*Supplementary Table S2* Estimation of mediation effects of poor mental health on poor sleep quality and insomnia.

| **Exposure** | **Mediator** | **PSQI score ≥ 6** | | **ISI score ≥ 8** | |
| --- | --- | --- | --- | --- | --- |
|  |  | **Percentage Mediated** | **p value** | **Percentage Mediated** | **p value** |
| Cumulative night work duration ^a^ | Depression | -3.5 (13.9) | 0.800 | -14.1 (63.1) | 0.823 |
|  | Anxiety | 7.8 (14.3) | 0.587 | 34.2 (58.4) | 0.558 |
| Morning shift start time ^b^ | Depression | -3.2 (17.1) | 0.853 | -13.2 (82.4) | 0.873 |
|  | Anxiety | -13.1 (21.6) | 0.545 | -68.5 (208.4) | 0.742 |
| Afternoon shift start time ^c^ | Depression | 57 (121.7) | 0.639 | 319.7 (2613.2) | 0.903 |
|  | Anxiety | 30.6 (80.0) | 0.702 | 146 (852.4) | 0.864 |
| Night shift start time ^d^ | Depression | 113.1 (249.2) | 0.650 | 61.8 (48.2) | 0.200 |
|  | Anxiety | 115.2 (250.7) | 0.646 | 59.6 (45.2) | 0.187 |
| Morning shift end time ^e^ | Depression | -36.4 (55.4) | 0.511 | -223.8 (1210.3) | 0.853 |
|  | Anxiety | -38.3 (61.2) | 0.532 | -365.8 (2801) | 0.896 |
| Afternoon shift end time ^f^ | Depression | 35.9 (37.5) | 0.339 | 49.4 (44.6) | 0.268 |
|  | Anxiety | 30.9 (37.2) | 0.406 | 41.0 (42.0) | 0.329 |
| Night shift end time ^g^ | Depression | 47.5 (110.7) | 0.668 | 335.3 (54197.0) | 0.995 |
|  | Anxiety | 85.4 (204.5) | 0.676 | 79.9 (3485.2) | 0.982 |

Abbreviation: PSQI, Pittsburg Sleep Quality Index; ISI, Insomnia Severity Index.

^a^ Model 3: adjusted for age, sex, cumulative years of night work, number of night shifts in the previous month, chronotype, Hospital anxiety and Depression Scale subscale score ≥ 8 (yes, no), whether people living in the same household smoked (yes or no), the habit of eating after 10 pm (yes or no), napping habit on workfree days (yes or no), noise level at home (quiet, relatively quiet, relatively noisy, noisy).

^b^ Model 4: adjusted for age, sex, morning shift start time, morning shift working hours, number of morning shifts in the previous month, chronotype, Hospital anxiety and Depression Scale subscale score ≥ 8 (yes, no), whether people living in the same household smoked (yes or no), the habit of eating after 10 pm (yes or no), napping habit on workfree days (yes or no), noise level at home (quiet, relatively quiet, relatively noisy, noisy).

^c^ Model 5: adjusted for age, sex, afternoon shift start time, afternoon shift working hours, number of afternoon shifts in the previous month, chronotype, Hospital anxiety and Depression Scale subscale score ≥ 8 (yes, no), whether people living in the same household smoked (yes or no), the habit of eating after 10 pm (yes or no), napping habit on workfree days (yes or no), noise level at home (quiet, relatively quiet, relatively noisy, noisy).

^e^ Model 6: adjusted for age, sex, night shift start time, night shift working hours, number of night shifts in the previous month, chronotype, Hospital anxiety and Depression Scale subscale score ≥ 8 (yes, no), whether people living in the same household smoked (yes or no), the habit of eating after 10 pm (yes or no), napping habit on workfree days (yes or no), noise level at home (quiet, relatively quiet, relatively noisy, noisy).

^d^ Model 7: adjusted for age, sex, morning shift end time, morning shift working hours, number of morning shifts in the previous month, chronotype, Hospital anxiety and Depression Scale subscale score ≥ 8 (yes, no), whether people living in the same household smoked (yes or no), the habit of eating after 10 pm (yes or no), napping habit on workfree days (yes or no), noise level at home (quiet, relatively quiet, relatively noisy, noisy).

^e^ Model 8: adjusted for age, sex, afternoon shift end time, afternoon shift working hours, number of afternoon shifts in the previous month, chronotype, Hospital anxiety and Depression Scale subscale score ≥ 8 (yes, no), whether people living in the same household smoked (yes or no), the habit of eating after 10 pm (yes or no), napping habit on workfree days (yes or no), noise level at home (quiet, relatively quiet, relatively noisy, noisy).

^f^ Model 9: adjusted for age, sex, night shift end time, night shift working hours, number of night shifts in the previous month, chronotype, Hospital anxiety and Depression Scale subscale score ≥ 8 (yes, no), whether people living in the same household smoked (yes or no), the habit of eating after 10 pm (yes or no), napping habit on workfree days (yes or no), noise level at home (quiet, relatively quiet, relatively noisy, noisy).

*Supplementary Table S3* The joint effect of work schedule and chronotype on sleep quality.

| **Chronotype** | **Work schedule/night work exposure** | **PSQI score ≥ 6** | | **ISI score ≥ 8** | |
| --- | --- | --- | --- | --- | --- |
|  |  | **n1/n2^a^** | **OR (95% CI) ^b^** | **n1/n2^a^** | **OR (95% CI)** |
| **Work schedules ^c^** | | | | | |
| **Morning type** | **Day work** | 19/15 | Ref. | 17/17 | Ref. |
|  | **Night shift work** | 178/65 | **2.58 (1.15, 5.78)** | 135/108 | 0.87 (0.39, 1.91) |
|  | **Other shift work ^d^** | 6/4 | 1.01 (0.23, 4.39) | 4/6 | 0.50 (0.11, 2.35) |
| **Intermediate type** | **Day work** | 46/52 | Ref. | 31/67 | Ref. |
|  | **Night shift work** | 254/40 | **4.95 (2.85, 8.59)** | 162/132 | **1.86 (1.05, 3.30)** |
|  | **Other shift work ^d^** | 13/12 | 1.08 (0.43, 2.73) | 7/18 | 0.69 (0.24, 1.97) |
| **Evening type** | **Day work** | 48/28 | Ref. | 29/47 | Ref. |
|  | **Night shift work** | 117/21 | **3.00 (1.58, 5.71)** | 84/54 | **2.53 (1.31, 4.88)** |
|  | **Other shift work ^d^** | 5/3 | 1.43 (0.31, 6.56) | 3/5 | 1.03 (0.19, 5.55) |
| **Work start time** | | | | | |
| **Morning shift/day work ^d^** | | | | | |
| **Morning type** | **< 7 a.m.** | 25/8 | 2.62 (0.40, 17.28) | 19/14 | 0.81 (0.12, 5.41) |
|  | **7 -< 8 a.m.** | 153/57 | 3.35 (0.59, 18.95) | 119/91 | 1.08 (0.19, 6.20) |
|  | **8 -< 9 a.m.** | 19/13 | 1.42 (0.30, 6.68) | 12/20 | 0.40 (0.09, 1.85) |
|  | **>- 9 a.m.** | 6/5 | Ref. | 6/5 | Ref. |
|  | **p for trend** |  | 0.114 |  | 0.083 |
| **Intermediate type** | **< 7 a.m.** | 19/4 | 2.52 (0.55, 11.56) | 12/11 | 2.48 (0.50, 12.28) |
|  | **7 -< 8 a.m.** | 232/40 | 1.90 (0.54, 6.68) | 150/122 | 3.18 (0.83, 12.21) |
|  | **8 -< 9 a.m.** | 40/35 | 1.10 (0.50, 2.44) | 24/51 | 0.92 (0.38, 2.25) |
|  | **>- 9 a.m.** | 22/25 | Ref. | 14/33 | Ref. |
|  | **p for trend** |  | 0.287 |  | 0.072 |
| **Evening type** | **< 7 a.m.** | 12/3 | 0.61 (0.08, 4.53) | 5/10 | 0.15 (0.02, 1.21) |
|  | **7 -< 8 a.m.** | 101/18 | 1.11 (0.22, 5.55) | 74/45 | 0.30 (0.06, 1.58) |
|  | **8 -< 9 a.m.** | 24/15 | 1.03 (0.41, 2.59) | 16/23 | 1.32 (0.49, 3.56) |
|  | **>- 9 a.m.** | 33/16 | Ref. | 21/28 | Ref. |
|  | **p for trend** |  | 0.502 |  | 0.032 |
| **Afternoon shift ^e^** | | | | | |
| **Morning type** | **<- 12 p.m.** | 0/3 | - | 1/2 | 0.44 (0.03, 6.38) |
|  | **12 >-1 p.m.** | 57/28 | Ref. | 40/45 | Ref. |
|  | **1 >-2 p.m.** | 102/21 | **2.28 (1.07, 4.86)** | 76/47 | 1.36 (0.63, 2.91) |
|  | **> 2 p.m.** | 20/16 | 0.61 (0.24, 1.55) | 20/16 | 0.76 (0.30, 1.94) |
|  | **p for trend** |  | 0.987 |  | 0.236 |
| **Intermediate type** | **<- 12 p.m.** | 3/3 | 0.20 (0.03, 1.29) | 0/6 | - |
|  | **12 >-1 p.m.** | 105/11 | Ref. | 71/45 | Ref. |
|  | **1 >-2 p.m.** | 107/22 | 1.22 (0.59, 2.52) | 67/62 | 1.16 (0.56, 2.40) |
|  | **> 2 p.m.** | 38/8 | 1.07 (0.46, 2.50) | 24/22 | 0.98 (0.41, 2.31) |
|  | **p for trend** |  | 0.071 |  | 0.983 |
| **Evening type** | **<- 12 p.m.** | 3/0 | 0.27 (0.02, 3.86) | 1/2 | **0.02 (0.01, 0.42)** |
|  | **12 >-1 p.m.** | 41/8 | Ref. | 29/20 | Ref. |
|  | **1 >-2 p.m.** | 48/11 | 1.09 (0.32, 3.77) | 35/24 | 1.48 (0.41, 5.40) |
|  | **> 2 p.m.** | 26/2 | 1.61 (0.40, 6.47) | 18/10 | 2.34 (0.55, 10.06) |
|  | **p for trend** |  | 0.198 |  | **0.010** |
| **Night shift ^f^** | | | | | |
| **Morning type** | **< 9 p.m.** | 17/9 | 0.59 (0.24, 1.48) | 13/13 | 0.65 (0.26, 1.61) |
|  | **9 -< 9.30 p.m.** | 91/25 | Ref. | 66/50 | Ref. |
|  | **9.30 -< 10 p.m.** | 43/15 | 0.89 (0.47, 1.70) | 33/25 | 1.48 (0.41, 5.40) |
|  | **>- 10 p.m.** | 27/16 | 0.41 (0.18, 0.95) | 23/20 | 2.34 (0.55, 10.06) |
|  | **p for trend** |  | 0.116 |  | 0.408 |
| **Intermediate type** | **< 9 p.m.** | 15/2 | 1.89 (0.59, 6.08) | 7/10 | 0.46 (0.14, 1.57) |
|  | **9 -< 9.30 p.m.** | 106/21 | Ref. | 66/61 | Ref. |
|  | **9.30 -< 10 p.m.** | 87/10 | 1.32 (0.74, 2.37) | 64/33 | 1.12 (0.59, 2.12) |
|  | **>- 10 p.m.** | 46/7 | 1.05 (0.49, 2.24) | 25/28 | 0.51 (0.17, 1.54) |
|  | **p for trend** |  | 0.411 |  | **0.037** |
| **Evening type** | **< 9 p.m.** | 12/3 | 1.11 (0.25, 4.95) | 6/9 | 0.57 (0.10, 3.22) |
|  | **9 -< 9.30 p.m.** | 41/11 | Ref. | 29/23 | Ref. |
|  | **9.30 -< 10 p.m.** | 36/3 | **2.88 (1.14, 7.30)** | 27/12 | **3.51 (1.33, 9.24)** |
|  | **>- 10 p.m.** | 28/4 | 2.12 (0.75, 5.96) | 22/10 | **3.40 (1.15, 10.07)** |
|  | **p for trend** |  | 0.205 |  | 0.077 |
| **Off-work time** | | | | | |
| **Morning shift/day work ^g^** | | | | | |
| **Morning type** | **< 3 p.m.** | 79/15 | Ref. | 59/35 | Ref. |
|  | **3 -< 3.30 p.m.** | 39/18 | **0.43 (0.19, 0.99)** | 35/22 | 1.06 (0.46, 2.42) |
|  | **3.30 -< 4 p.m.** | 49/30 | 0.25 (0.08, 0.75) | 35/44 | 0.51 (0.17, 1.55) |
|  | **>- 4 p.m.** | 36/20 | 0.31 (0.07, 1.30) | 27/29 | 0.83 (0.20, 3.49) |
|  | **p for trend** |  | 0.286 |  | 0.116 |
| **Intermediate type** | **< 3 p.m.** | 71/21 | Ref. | 47/45 | Ref. |
|  | **3 -< 3.30 p.m.** | 48/10 | 0.98 (0.47, 2.06) | 26/32 | 0.80 (0.37, 1.72) |
|  | **3.30 -< 4 p.m.** | 117/10 | 1.31 (0.60, 2.90) | 78/49 | 1.21 (0.53, 2.77) |
|  | **>- 4 p.m.** | 77/63 | 1.44 (0.48, 4.34) | 49/91 | 0.90 (0.29, 2.86) |
|  | **p for trend** |  | 0.458 |  | 0.272 |
| **Evening type** | **< 3 p.m.** | 30/8 | Ref. | 18/20 | Ref. |
|  | **3 -< 3.30 p.m.** | 32/5 | 1.29 (0.47, 3.58) | 23/14 | 1.84 (0.66, 5.16) |
|  | **3.30 -< 4 p.m.** | 47/7 | 1.76 (0.57, 5.39) | 35/19 | 2.46 (0.77, 7.85) |
|  | **>- 4 p.m.** | 61/32 | 0.86 (0.18, 4.05) | 40/53 | 2.36 (0.47, 11.72) |
|  | **p for trend** |  | 0.247 |  | 0.722 |
| **Afternoon shift ^h^** | | | | | |
| **Morning type** | **<- 9 p.m.** | 15/10 | 0.29 (0.11, 0.74) | 13/12 | 0.64 (0.25, 1.63) |
|  | **9 <- 9.30 p.m.** | 71/13 | Ref. | 52/32 | Ref. |
|  | **9.30 <- 10 p.m.** | 69/31 | 0.61 (0.29, 1.27) | 51/49 | 0.97 (0.46, 2.05) |
|  | **> 10 p.m.** | 24/14 | 0.47 (0.20, 1.11) | 21/17 | 0.78 (0.33, 1.86) |
|  | **p for trend** |  | 0.196 |  | 0.426 |
| **Intermediate type** | **<- 9 p.m.** | 18/9 | 0.47 (0.18, 1.21) | 13/14 | 0.55 (0.21, 1.44) |
|  | **9 <- 9.30 p.m.** | 70/15 | Ref. | 43/42 | Ref. |
|  | **9.30 <- 10 p.m.** | 110/13 | 0.80 (0.37, 1.69) | 71/52 | 0.86 (0.41, 1.83) |
|  | **> 10 p.m.** | 55/7 | 0.93 (0.42, 2.04) | 35/27 | 0.92 (0.42, 2.02) |
|  | **p for trend** |  | 0.187 |  | 0.322 |
| **Evening type** | **<- 9 p.m.** | 11/3 | 0.78 (0.20, 3.06) | 5/9 | 0.39 (0.08, 1.85) |
|  | **9 <- 9.30 p.m.** | 28/7 | Ref. | 18/17 | Ref. |
|  | **9.30 <- 10 p.m.** | 49/7 | **3.67 (1.23, 10.91)** | 37/19 | **3.57 (1.12, 11.40)** |
|  | **> 10 p.m.** | 30/4 | 2.10 (0.73, 6.07) | 23/11 | 2.93 (0.94, 9.17) |
|  | **p for trend** |  | **0.045** |  | **0.013** |
| **Night shift ^i^** | | | | | |
| **Morning type** | **< 7 a.m.** | 13/5 | 0.60 (0.22, 1.63) | 10/8 | 0.71 (0.26, 1.92) |
|  | **7 -< 7.15 a.m.** | 99/36 | Ref. | 79/56 | Ref. |
|  | **7.15 -< 7.30 a.m.** | 41/16 | 0.77 (0.41, 1.46) | 27/30 | 0.52 (0.27, 1.00) |
|  | **>- 7.30 a.m.** | 25/8 | 0.96 (0.44, 2.10) | 19/14 | 0.77 (0.35, 1.71) |
|  | **p for trend** |  | 0.434 |  | 0.386 |
| **Intermediate type** | **< 7 a.m.** | 8/1 | 2.36 (0.57, 9.71) | 4/5 | 0.46 (0.11, 1.93) |
|  | **7 -< 7.15 a.m.** | 130/22 | Ref. | 82/70 | Ref. |
|  | **7.15 -< 7.30 a.m.** | 78/14 | 1.08 (0.62, 1.88) | 54/38 | 0.98 (0.56, 1.72) |
|  | **>- 7.30 a.m.** | 38/3 | 1.10 (0.50, 2.42) | 22/19 | 0.98 (0.44, 2.16) |
|  | **p for trend** |  | 0.333 |  | 0.342 |
| **Evening type** | **< 7 a.m.** | 4/3 | 0.22 (0.04, 1.33) | 1/6 | 0.16 (0.02, 1.76) |
|  | **7 -< 7.15 a.m.** | 61/12 | Ref. | 47/26 | Ref. |
|  | **7.15 -< 7.30 a.m.** | 34/5 | 1.17 (0.51, 2.68) | 24/15 | 1.44 (0.62, 3.39) |
|  | **>- 7.30 a.m.** | 18/1 | 1.73 (0.55, 5.49) | 12/7 | 1.05 (0.32, 3.44) |
|  | **p for trend** |  | 0.051 |  | 0.089 |

Abbreviation: OR, odds ratio; CI, confidence interval; Ref., reference; PSQI, Pittsburg Sleep Quality Index; ISI, Insomnia Severity Index.

^a^ n1/n2: Number of cases over number of non-cases.

^b^ ORs of multivariate adjusted models.

^c^ Model 2: adjusted for age, sex, work schedule (daytime work, night shift work, other shift work), chronotype (morning type, intermediate type, evening type), Hospital anxiety and Depression Scale subscale score ≥ 8 (yes, no), whether people living in the same household smoked (yes or no), the habit of eating after 10 pm (yes or no), napping habit on workfree days (yes or no), years of working (0 <- 1 year, 1 <- 5 years, 5 <- 10 years, >10 years), noise level at home (quiet, relatively quiet, relatively noisy, noisy).

^d^ Model 4: adjusted for age, sex, morning shift start time, morning shift working hours, number of morning shifts in the previous month, chronotype, Hospital anxiety and Depression Scale subscale score ≥ 8 (yes, no), whether people living in the same household smoked (yes or no), the habit of eating after 10 pm (yes or no), napping habit on workfree days (yes or no), noise level at home (quiet, relatively quiet, relatively noisy, noisy)

^e^ Model 5: adjusted for age, sex, afternoon shift start time, afternoon shift working hours, number of afternoon shifts in the previous month, chronotype, Hospital anxiety and Depression Scale subscale score ≥ 8 (yes, no), whether people living in the same household smoked (yes or no), the habit of eating after 10 pm (yes or no), napping habit on workfree days (yes or no), noise level at home (quiet, relatively quiet, relatively noisy, noisy).

^f^ Model 6: adjusted for age, sex, night shift start time, night shift working hours, number of night shifts in the previous month, chronotype, Hospital anxiety and Depression Scale subscale score ≥ 8 (yes, no), whether people living in the same household smoked (yes or no), the habit of eating after 10 pm (yes or no), napping habit on workfree days (yes or no), noise level at home (quiet, relatively quiet, relatively noisy, noisy).

^g^ Model 7: adjusted for age, sex, morning shift end time, morning shift working hours, number of morning shifts in the previous month, chronotype, Hospital anxiety and Depression Scale subscale score ≥ 8 (yes, no), whether people living in the same household smoked (yes or no), the habit of eating after 10 pm (yes or no), napping habit on workfree days (yes or no), noise level at home (quiet, relatively quiet, relatively noisy, noisy).

^h^ Model 8: adjusted for age, sex, afternoon shift end time, afternoon shift working hours, number of afternoon shifts in the previous month, chronotype, Hospital anxiety and Depression Scale subscale score ≥ 8 (yes, no), whether people living in the same household smoked (yes or no), the habit of eating after 10 pm (yes or no), napping habit on workfree days (yes or no), noise level at home (quiet, relatively quiet, relatively noisy, noisy).

*Supplementary Table S4* The main effect of work schedule, chronotype, night work exposure on sleep quality and insomnia by removing those who smoked (n=15), with chronic diseases (n=96), direct family members who had mental disorders (n=100).

| **Work schedule/night work exposure** | **PSQI score ≥ 6** | | | **ISI score ≥ 8** | | |
| --- | --- | --- | --- | --- | --- | --- |
|  | **n1/n2^a^** | **OR (95% CI) ^b^** | **OR (95% CI) ^c^** | **n1/n2^a^** | **OR (95% CI) ^b^** | **OR (95% CI)** |
| **Work schedule ^d^** | | | | | | |
| **Day work** | 88/79 | Ref. | Ref. | 59/108 | Ref. | Ref. |
| **Night shift work** | 433/104 | 4.01 (2.82, 5.71) | **3.56 (2.39, 5.30)** | 298/239 | 2.39 (1.67, 3.41) | **1.87 (1.24, 2.83)** |
| **Other shift work** | 20/16 | 1.26 (0.62, 2.56) | 1.34 (0.64, 2.81) | 10/26 | 0.80 (0.36, 1.77) | 0.71 (0.30, 1.66) |
| **Chronotype ^d^** | | | | | | |
| **Morning type** | 162/74 | Ref. | Ref. | 125/111 | Ref. | Ref. |
| **Intermediate type** | 250/81 | 1.22 (0.88, 1.69) | **1.44 (1.02, 2.04)** | 157/174 | 0.79 (0.57, 1.09) | 0.77 (0.54, 1.09) |
| **Evening type** | 129/44 | 1.14 (0.78, 1.67) | **1.70 (1.12, 2.58)** | 85/88 | 0.77 (0.52, 1.12) | 0.89 (0.59, 1.36) |
| **Cumulative night work duration ^e^** | | | | | | |
| **No night work** | 89/79 | Ref. | Ref. | 59/109 | Ref. | Ref. |
| **0 <- 5 Years** | 164/42 | 2.67 (1.73, 4.12) | 1.09 (0.50, 2.39) | 114/92 | 1.91 (1.23, 2.95) | 0.75 (0.31, 1.81) |
| **5 <- 10 Years** | 184/54 | 3.89 (2.58, 5.85) | 1.56 (0.72, 3.40) | 122/116 | 2.15 (1.42, 3.24) | 0.82 (0.34, 1.98) |
| **> 10 Years** | 104/24 | 5.41 (3.30, 8.87) | 2.06 (0.86, 4.90) | 72/56 | 3.29 (2.01, 5.39) | 1.28 (0.49, 3.34) |
| **p for trend** |  | 0.010 | **0.028** |  | 0.045 | 0.067 |
| **Number of night work last month ^e^** | | | | | | |
| **No night work** | 108/95 | Ref. | Ref. | 69/134 | Ref. | Ref. |
| **1-3 nights** | 113/29 | 3.29 (2.14, 5.07) | 1.96 (0.90, 4.27) | 77/65 | 2.27 (1.47, 3.48) | 1.83 (0.77, 4.36) |
| **4-6 nights** | 293/73 | 3.99 (2.81, 5.68) | **2.76 (1.33, 5.73)** | 208/158 | 2.57 (1.81, 3.66) | **2.49 (1.09, 5.66)** |
| **>- 7 nights** | 27/2 | 5.41 (2.59, 11.34) | **3.61 (1.31, 9.93)** | 13/16 | 2.31 (1.12, 4.76) | **3.61 (1.31, 9.93)** |
| **p for trend** |  | 0.206 | 0.154 |  | 0.552 | 0.166 |
| **Work start time** | | | | | | |
| **Morning shift ^f^** | | | | | | |
| **< 7 a.m.** | 41/10 | 2.92 (1.48, 5.78) | 1.86 (0.66, 5.25) | 27/24 | 1.87 (0.94, 3.70) | 0.91 (0.30, 2.70) |
| **7 -< 8 a.m.** | 387/95 | 3.65 (2.31, 5.77) | 1.95 (0.82, 4.65) | 267/215 | 2.21 (1.38, 3.54) | 1.01 (0.40, 2.57) |
| **8 -< 9 a.m.** | 66/54 | 0.99 (0.58, 1.69) | 1.13 (0.64, 2.02) | 41/79 | 0.98 (0.55, 1.74) | 1.04 (0.55, 1.95) |
| **>- 9 a.m.** | 47/39 | Ref. | Ref. | 32/54 | Ref. | Ref. |
| **p for trend** |  | <0.0001 | 0.249 |  | 0.009 | 0.797 |
| **Afternoon shift ^g^** | | | | | | |
| **<- 12 p.m.** | 5/6 | 0.19 (0.06, 0.66) | **0.13 (0.03, 0.51)** | 2/9 | 0.20 (0.04, 0.95) | **0.17 (0.03, 0.92)** |
| **12 <- 1 p.m.** | 160/42 | Ref. | Ref. | 107/95 | Ref. | Ref. |
| **1 <- 2 p.m.** | 203/41 | 1.34 (0.93, 1.93) | **1.72 (1.04, 2.83)** | 139/105 | 1.18 (0.83, 1.69) | 1.32 (0.80, 2.18) |
| **> 2 p.m.** | 66/20 | 0.91 (0.55, 1.50) | 1.02 (0.56, 1.85) | 49/37 | 1.24 (0.76, 2.01) | 1.17 (0.64, 2.12) |
| **p for trend** |  | 0.001 | **< 0.001** |  | 0.024 | **0.020** |
| **Night shift ^h^** | | | | | | |
| **< 9 p.m.** | 33/11 | Ref. | Ref. | 19/25 | Ref. | Ref. |
| **9 -< 9.30 p.m.** | 180/45 | 1.29 (0.69, 2.44) | 1.18 (0.58, 2.39) | 121/104 | 1.49 (0.79, 2.82) | 1.45 (0.69, 3.03) |
| **9.30 -< 10 p.m.** | 139/27 | 1.93 (1.00, 3.73) | 1.40 (0.66, 2.98) | 102/64 | 1.96 (1.02, 3.76) | 1.64 (0.75, 3.58) |
| **>- 10 p.m.** | 81/21 | 1.29 (0.65, 2.59) | 0.96 (0.40, 2.28) | 56/46 | 1.79 (0.89, 3.57) | 1.56 (0.64, 3.81) |
| **p for trend** |  | 0.037 | 0.163 |  | 0.333 | 0.659 |
| **Off-work time** | | | | | | |
| **Morning shift ^i^** | | | | | | |
| **< 3 p.m.** | 135/34 | Ref. | Ref. | 95/74 | Ref. | Ref. |
| **3 -< 3.30 p.m.** | 93/24 | 0.85 (0.53, 1.34) | 0.77 (0.45, 1.29) | 64/53 | 0.94 (0.6, 1.47) | 0.99 (0.59, 1.68) |
| **3.30 -< 4 p.m.** | 171/42 | 0.84 (0.57, 1.26) | 0.74 (0.41, 1.31) | 115/98 | 0.84 (0.57, 1.23) | 0.85 (0.47, 1.53) |
| **>- 4 p.m.** | 142/98 | 0.33 (0.22, 0.49) | 0.76 (0.34, 1.68) | 93/147 | 0.50 (0.34, 0.73) | 0.97 (0.43, 2.19) |
| **p for trend** |  | <0.001 | 0.879 |  | 0.004 | 0.524 |
| **Afternoon shift ^j^** | | | | | | |
| **<- 9 p.m.** | 34/19 | Ref. | Ref. | 25/28 | Ref. | Ref. |
| **9 <- 9.30 p.m.** | 131/29 | 2.25 (1.22, 4.15) | **2.36 (1.24, 4.48)** | 88/72 | 1.35 (0.74, 2.46) | 1.47 (0.76, 2.82) |
| **9.30 <- 10 p.m.** | 179/42 | 2.23 (1.24, 4.03) | **2.31 (1.16, 4.60)** | 119/102 | 1.31 (0.73, 2.34) | 1.52 (0.75, 3.08) |
| **> 10 p.m.** | 90/19 | 2.38 (1.25, 4.54) | **2.33 (1.15, 4.75)** | 65/44 | 1.66 (0.88, 3.13) | 1.81 (0.88, 3.73) |
| **p for trend** |  | 0.814 | 0.939 |  | 0.372 | 0.438 |
| **Night shift ^k^** | | | | | | |
| **< 7 a.m.** | 20/7 | Ref. | Ref. | 13/14 | Ref. | Ref. |
| **7 -< 7.15 a.m.** | 223/53 | 1.32 (0.61, 2.85) | 1.41 (0.63, 3.17) | 158/118 | 1.29 (0.60, 2.76) | 1.78 (0.77, 4.08) |
| **7.15 -< 7.30 a.m.** | 120/32 | 1.34 (0.60, 2.98) | 1.09 (0.47, 2.53) | 80/72 | 1.11 (0.50, 2.43) | 1.23 (0.52, 2.93) |
| **>- 7.30 a.m.** | 70/12 | 1.49 (0.63, 3.49) | 1.25 (0.51, 3.07) | 47/35 | 1.36 (0.59, 3.13) | 1.72 (0.69, 4.30) |
| **p for trend** |  | 0.622 | 0.340 |  | 0.359 | 0.101 |

Abbreviation: OR, odds ratio; CI, confidence interval; Ref., reference; PSQI, Pittsburg Sleep Quality Index; ISI, Insomnia Severity Index.

^a^ n1/n2: Number of cases over number of non-cases.

^b^ Model 1: adjusted for age and sex.

^c^ ORs of adjusted models.

^d^ Model 2: additionally adjusted for work schedule (daytime work, night shift work, other shift work), chronotype (morning type, intermediate type, evening type), Hospital anxiety and Depression Scale subscale score ≥ 8 (yes, no), whether people living in the same household smoked (yes or no), the habit of eating after 10 pm (yes or no), napping habit on workfree days (yes or no), years of working (0 <- 1 year, 1 <- 5 years, 5 <- 10 years, >10 years), noise level at home (quiet, relatively quiet, relatively noisy, noisy).

^e^ Model 3: adjusted for age and sex, adjusted for age, sex, cumulative years of night work, number of night shifts in the previous month, chronotype, Hospital anxiety and Depression Scale subscale score ≥ 8 (yes, no), whether people living in the same household smoked (yes or no), the habit of eating after 10 pm (yes or no), napping habit on workfree days (yes or no), noise level at home (quiet, relatively quiet, relatively noisy, noisy).

^f^ Model 4: adjusted for age, sex, morning shift start time, morning shift working hours, number of morning shifts in the previous month, chronotype, Hospital anxiety and Depression Scale subscale score ≥ 8 (yes, no), whether people living in the same household smoked (yes or no), the habit of eating after 10 pm (yes or no), napping habit on workfree days (yes or no), noise level at home (quiet, relatively quiet, relatively noisy, noisy).

^g^ Model 5: adjusted for age, sex, afternoon shift start time, afternoon shift working hours, number of afternoon shifts in the previous month, chronotype, Hospital anxiety and Depression Scale subscale score ≥ 8 (yes, no), whether people living in the same household smoked (yes or no), the habit of eating after 10 pm (yes or no), napping habit on workfree days (yes or no), noise level at home (quiet, relatively quiet, relatively noisy, noisy).

^h^ Model 6: adjusted for age, sex, night shift start time, night shift working hours, number of night shifts in the previous month, chronotype, Hospital anxiety and Depression Scale subscale score ≥ 8 (yes, no), whether people living in the same household smoked (yes or no), the habit of eating after 10 pm (yes or no), napping habit on workfree days (yes or no), noise level at home (quiet, relatively quiet, relatively noisy, noisy).

^i^ Model 7: adjusted for age, sex, morning shift end time, morning shift working hours, number of morning shifts in the previous month, chronotype, Hospital anxiety and Depression Scale subscale score ≥ 8 (yes, no), whether people living in the same household smoked (yes or no), the habit of eating after 10 pm (yes or no), napping habit on workfree days (yes or no), noise level at home (quiet, relatively quiet, relatively noisy, noisy).

^j^ Model 8: adjusted for age, sex, afternoon shift end time, afternoon shift working hours, number of afternoon shifts in the previous month, chronotype, Hospital anxiety and Depression Scale subscale score ≥ 8 (yes, no), whether people living in the same household smoked (yes or no), the habit of eating after 10 pm (yes or no), napping habit on workfree days (yes or no), noise level at home (quiet, relatively quiet, relatively noisy, noisy).

^k^ Model 9: adjusted for age, sex, night shift end time, night shift working hours, number of night shifts in the previous month, chronotype, Hospital anxiety and Depression Scale subscale score ≥ 8 (yes, no), whether people living in the same household smoked (yes or no), the habit of eating after 10 pm (yes or no), napping habit on workfree days (yes or no), noise level at home (quiet, relatively quiet, relatively noisy, noisy).

*Supplementary Table S5* The association between core exposure variables and poor sleep quality and insomnia by removing outpatient (n=53) and emergency room (n=33) nurses.

| **Work schedule/night work exposure** | **PSQI score ≥ 6** | | | **ISI score ≥ 8** | | |
| --- | --- | --- | --- | --- | --- | --- |
|  | **n1/n2^a^** | **OR (95% CI) ^b^** | **OR (95% CI) ^c^** | **n1/n2^a^** | **OR (95% CI) ^b^** | **OR (95% CI)** |
| **Work schedule ^d^** | | | | | | |
| **Day work** | 113/95 | Ref. | Ref. | 77/131 | Ref. | Ref. |
| **Night shift work** | 496/116 | 3.72 (2.69, 5.13) | 3.33 (2.32, 4.78) | 343/269 | 2.17 (1.58, 2.99) | 1.60 (1.10, 2.32) |
| **Other shift work** | 8/12 | 0.61 (0.24, 1.52) | 0.73 (0.28, 1.88) | 6/14 | 0.67 (0.24, 1.82) | 0.59 (0.19, 1.79) |
| **Chronotype ^d^** | | | | | | |
| **Morning type** | 180/79 | Ref. | Ref. | 140/119 | Ref. | Ref. |
| **Intermediate type** | 283/94 | 1.26 (0.92, 1.71) | 1.45 (1.05, 2.02) | 182/195 | 0.77 (0.57, 1.04) | 0.69 (0.50, 0.96) |
| **Evening type** | 154/50 | 1.24 (0.86, 1.77) | 1.68 (1.14, 2.48) | 104/100 | 0.82 (0.57, 1.17) | 0.86 (0.58, 1.27) |
| **Cumulative night work duration ^e^** | | | | | | |
| **No night work** | 114/95 | Ref. | Ref. | 77/132 | Ref. | Ref. |
| **0 <- 5 Years** | 181/45 | 2.76 (1.82, 4.18) | 0.53 (0.19, 1.46) | 132/94 | 2.13 (1.41, 3.22) | 0.70 (0.22, 2.21) |
| **5 <- 10 Years** | 193/52 | 3.83 (2.60, 5.64) | 0.81 (0.29, 2.22) | 126/119 | 1.83 (1.25, 2.68) | 0.65 (0.21, 2.04) |
| **> 10 Years** | 129/31 | 3.93 (2.54, 6.09) | 0.68 (0.23, 1.95) | 91/69 | 2.51 (1.62, 3.87) | 0.76 (0.23, 2.49) |
| **p for trend** |  | 0.176 | 0.356 |  | 0.169 | 0.781 |
| **Number of night work last month ^e^** | | | | | | |
| **No night work** | 121/107 | Ref. | Ref. | 83/145 | Ref. | Ref. |
| **1-3 nights** | 117/34 | 3.05 (2.02, 4.60) | 3.88 (1.41, 10.72) | 82/69 | 2.07 (1.38, 3.11) | 2.09 (0.67, 6.51) |
| **4-6 nights** | 343/80 | 4.03 (2.89, 5.61) | 5.70 (2.13, 15.23) | 241/182 | 2.28 (1.65, 3.16) | 2.60 (0.86, 7.86) |
| **>- 7 nights** | 36/2 | 6.62 (3.35, 13.06) | 9.42 (2.95, 30.04) | 20/18 | 2.70 (1.39, 5.21) | 2.92 (0.83, 10.29) |
| **p for trend** |  | 0.031 | 0.018 |  | 0.448 | 0.377 |
| **Work start time** | | | | | | |
| **Morning shift ^f^** | | | | | | |
| **< 7 a.m.** | 53/15 | 2.57 (1.39, 4.73) | 1.82 (0.64, 5.19) | 34/34 | 1.72 (0.93, 3.19) | 1.32 (0.43, 4.06) |
| **7 -< 8 a.m.** | 441/106 | 3.59 (2.33, 5.53) | 2.23 (0.89, 5.61) | 311/236 | 2.35 (1.51, 3.67) | 1.85 (0.68, 5.04) |
| **8 -< 9 a.m.** | 70/58 | 0.96 (0.57, 1.60) | 1.16 (0.67, 2.02) | 46/82 | 1.05 (0.61, 1.81) | 1.26 (0.69, 2.31) |
| **>- 9 a.m.** | 53/43 | Ref. | Ref. | 35/61 | Ref. | Ref. |
| **p for trend** |  | 0.001 | 0.105 |  | 0.001 | 0.192 |
| **Afternoon shift ^g^** | | | | | | |
| **<- 12 p.m.** | 3/6 | 0.10 (0.02, 0.42) | 0.09 (0.02, 0.41) | 2/7 | 0.23 (0.05, 1.15) | 0.27 (0.05, 1.56) |
| **12 <- 1 p.m.** | 198/44 | Ref. | Ref. | 135/107 | Ref. | Ref. |
| **1 <- 2 p.m.** | 228/48 | 1.06 (0.76, 1.49) | 1.33 (0.82, 2.18) | 155/121 | 1.07 (0.77, 1.49) | 1.37 (0.83, 2.27) |
| **> 2 p.m.** | 69/23 | 0.82 (0.51, 1.31) | 0.91 (0.51, 1.64) | 54/38 | 1.21 (0.76, 1.92) | 1.31 (0.72, 2.39) |
| **p for trend** |  | 0.005 | **0.004** |  | 0.046 | **0.008** |
| **Night shift ^h^** | | | | | | |
| **< 9 p.m.** | 40/13 | Ref. | Ref. | 24/29 | Ref. | Ref. |
| **9 -< 9.30 p.m.** | 230/55 | 1.24 (0.70, 2.23) | 1.19 (0.63, 2.25) | 153/132 | 1.32 (0.74, 2.34) | 1.43 (0.74, 2.80) |
| **9.30 -< 10 p.m.** | 159/27 | 1.87 (1.02, 3.43) | 1.49 (0.75, 2.96) | 117/69 | 1.89 (1.04, 3.43) | 1.80 (0.88, 3.67) |
| **>- 10 p.m.** | 67/21 | 1.05 (0.53, 2.06) | 1.04 (0.46, 2.36) | 49/39 | 1.72 (0.89, 3.35) | 1.91 (0.82, 4.48) |
| **p for trend** |  | 0.009 | 0.622 |  | 0.249 | 0.297 |
| **Off-work time** | | | | | | |
| **Morning shift ^i^** | | | | | | |
| **< 3 p.m.** | 171/42 | Ref. | Ref. | 116/97 | Ref. | Ref. |
| **3 -< 3.30 p.m.** | 93/27 | 0.85 (0.55, 1.31) | 0.84 (0.51, 1.39) | 67/53 | 1.04 (0.68, 1.59) | 1.13 (0.68, 1.86) |
| **3.30 -< 4 p.m.** | 206/46 | 0.99 (0.69, 1.41) | 0.95 (0.55, 1.63) | 141/111 | 0.99 (0.70, 1.40) | 1.07 (0.62, 1.88) |
| **>- 4 p.m.** | 147/107 | 0.34 (0.24, 0.49) | 1.15 (0.50, 2.63) | 102/152 | 0.56 (0.39, 0.79) | 1.53 (0.65, 3.58) |
| **p for trend** |  | 0.001 | 0.426 |  | 0.004 | 0.433 |
| **Afternoon shift ^j^** | | | | | | |
| **<- 9 p.m.** | 40/20 | Ref. | Ref. | 30/30 | Ref. | Ref. |
| **9 <- 9.30 p.m.** | 163/34 | 2.03 (1.15, 3.60) | 2.23 (1.23, 4.05) | 107/90 | 1.15 (0.66, 2.00) | 1.43 (0.78, 2.64) |
| **9.30 <- 10 p.m.** | 207/46 | 2.11 (1.21, 3.67) | 2.18 (1.11, 4.29) | 143/110 | 1.25 (0.73, 2.15) | 1.47 (0.73, 2.93) |
| **> 10 p.m.** | 88/21 | 2.12 (1.14, 3.93) | 2.02 (1.02, 4.03) | 66/43 | 1.57 (0.86, 2.88) | 1.76 (0.87, 3.55) |
| **p for trend** |  | 0.863 | 0.712 |  | 0.163 | 0.438 |
| **Night shift ^k^** | | | | | | |
| **< 7 a.m.** | 24/9 | Ref. | Ref. | 14/19 | Ref. | Ref. |
| **7 -< 7.15 a.m.** | 257/63 | 1.51 (0.74, 3.05) | 1.44 (0.69, 2.99) | 183/137 | 1.6 (0.79, 3.23) | 2.07 (0.95, 4.47) |
| **7.15 -< 7.30 a.m.** | 150/34 | 1.95 (0.94, 4.05) | 1.46 (0.69, 3.13) | 101/83 | 1.52 (0.74, 3.14) | 1.59 (0.72, 3.53) |
| **>- 7.30 a.m.** | 65/10 | 1.83 (0.81, 4.11) | 1.64 (0.70, 3.82) | 45/30 | 1.81 (0.81, 4.02) | 2.35 (0.97, 5.68) |
| **p for trend** |  | 0.443 | 0.634 |  | 0.554 | 0.110 |

Abbreviation: OR, odds ratio; CI, confidence interval; Ref., reference; PSQI, Pittsburg Sleep Quality Index; ISI, Insomnia Severity Index.

^a^ n1/n2: Number of cases over number of non-cases.

^b^ Model 1: adjusted for age and sex.

^c^ ORs of adjusted models.

^d^ Model 2: additionally adjusted for work schedule (daytime work, night shift work, other shift work), chronotype (morning type, intermediate type, evening type), Hospital anxiety and Depression Scale subscale score ≥ 8 (yes, no), whether people living in the same household smoked (yes or no), the habit of eating after 10 pm (yes or no), napping habit on workfree days (yes or no), years of working (0 <- 1 year, 1 <- 5 years, 5 <- 10 years, >10 years), noise level at home (quiet, relatively quiet, relatively noisy, noisy).

^e^ Model 3: adjusted for age and sex, adjusted for age, sex, cumulative years of night work, number of night shifts in the previous month, chronotype, Hospital anxiety and Depression Scale subscale score ≥ 8 (yes, no), whether people living in the same household smoked (yes or no), the habit of eating after 10 pm (yes or no), napping habit on workfree days (yes or no), noise level at home (quiet, relatively quiet, relatively noisy, noisy).

^f^ Model 4: adjusted for age, sex, morning shift start time, morning shift working hours, number of morning shifts in the previous month, chronotype, Hospital anxiety and Depression Scale subscale score ≥ 8 (yes, no), whether people living in the same household smoked (yes or no), the habit of eating after 10 pm (yes or no), napping habit on workfree days (yes or no), noise level at home (quiet, relatively quiet, relatively noisy, noisy).

^g^ Model 5: adjusted for age, sex, afternoon shift start time, afternoon shift working hours, number of afternoon shifts in the previous month, chronotype, Hospital anxiety and Depression Scale subscale score ≥ 8 (yes, no), whether people living in the same household smoked (yes or no), the habit of eating after 10 pm (yes or no), napping habit on workfree days (yes or no), noise level at home (quiet, relatively quiet, relatively noisy, noisy).

^h^ Model 6: adjusted for age, sex, night shift start time, night shift working hours, number of night shifts in the previous month, chronotype, Hospital anxiety and Depression Scale subscale score ≥ 8 (yes, no), whether people living in the same household smoked (yes or no), the habit of eating after 10 pm (yes or no), napping habit on workfree days (yes or no), noise level at home (quiet, relatively quiet, relatively noisy, noisy).

^i^ Model 7: adjusted for age, sex, morning shift end time, morning shift working hours, number of morning shifts in the previous month, chronotype, Hospital anxiety and Depression Scale subscale score ≥ 8 (yes, no), whether people living in the same household smoked (yes or no), the habit of eating after 10 pm (yes or no), napping habit on workfree days (yes or no), noise level at home (quiet, relatively quiet, relatively noisy, noisy).

^j^ Model 8: adjusted for age, sex, afternoon shift end time, afternoon shift working hours, number of afternoon shifts in the previous month, chronotype, Hospital anxiety and Depression Scale subscale score ≥ 8 (yes, no), whether people living in the same household smoked (yes or no), the habit of eating after 10 pm (yes or no), napping habit on workfree days (yes or no), noise level at home (quiet, relatively quiet, relatively noisy, noisy).

^k^ Model 9: adjusted for age, sex, night shift end time, night shift working hours, number of night shifts in the previous month, chronotype, Hospital anxiety and Depression Scale subscale score ≥ 8 (yes, no), whether people living in the same household smoked (yes or no), the habit of eating after 10 pm (yes or no), napping habit on workfree days (yes or no), noise level at home (quiet, relatively quiet, relatively noisy, noisy).

*Supplementary Figure S1* Modelling flow diagrams.

1. Age and sex adjusted initial models.

+

Core exposure variables

Work schedules

Chronotype

Cumulative years of night work

Number of night shifts in the previous month

Specific timing parameters

Morning/afternoon/night shift start times

- Age
- Sex

Initial models (Model 1)

+

+

+

+

- Poor sleep quality (PSQI ≥ 6)
- Insomnia (ISI ≥ 6)

Outcomes

Morning/afternoon/night shift end times

+

1. Multivariate adjusted models.

- Age
- Sex
- HADS subscale score ≥ 8 (yes, no)
- Household smoking (yes, no)
- Late-night eating (yes, no)
- Napping behaviours (yes, no)
- Perceived home noise levels (quiet, relatively quiet, relatively noisy, noisy)

Confounding variables

Core exposure variables

Work schedules

Chronotype

Cumulative years of night work

Number of night shifts in the previous month

Specific timing parameters

Morning/afternoon/night shift start times

Morning/afternoon/night shift end times

Years of working

+

+

Model 2

Chronotype

+

+

Model 3

- Working hours
- Number of shifts in the previous month
- Chronotype

+

+

Models 4-9

+

+

- Poor sleep quality (PSQI ≥ 6)
- Insomnia (ISI ≥ 6)

Outcomes

*Supplementary Figure* 2 Preliminary data cleaning process of baseline sample


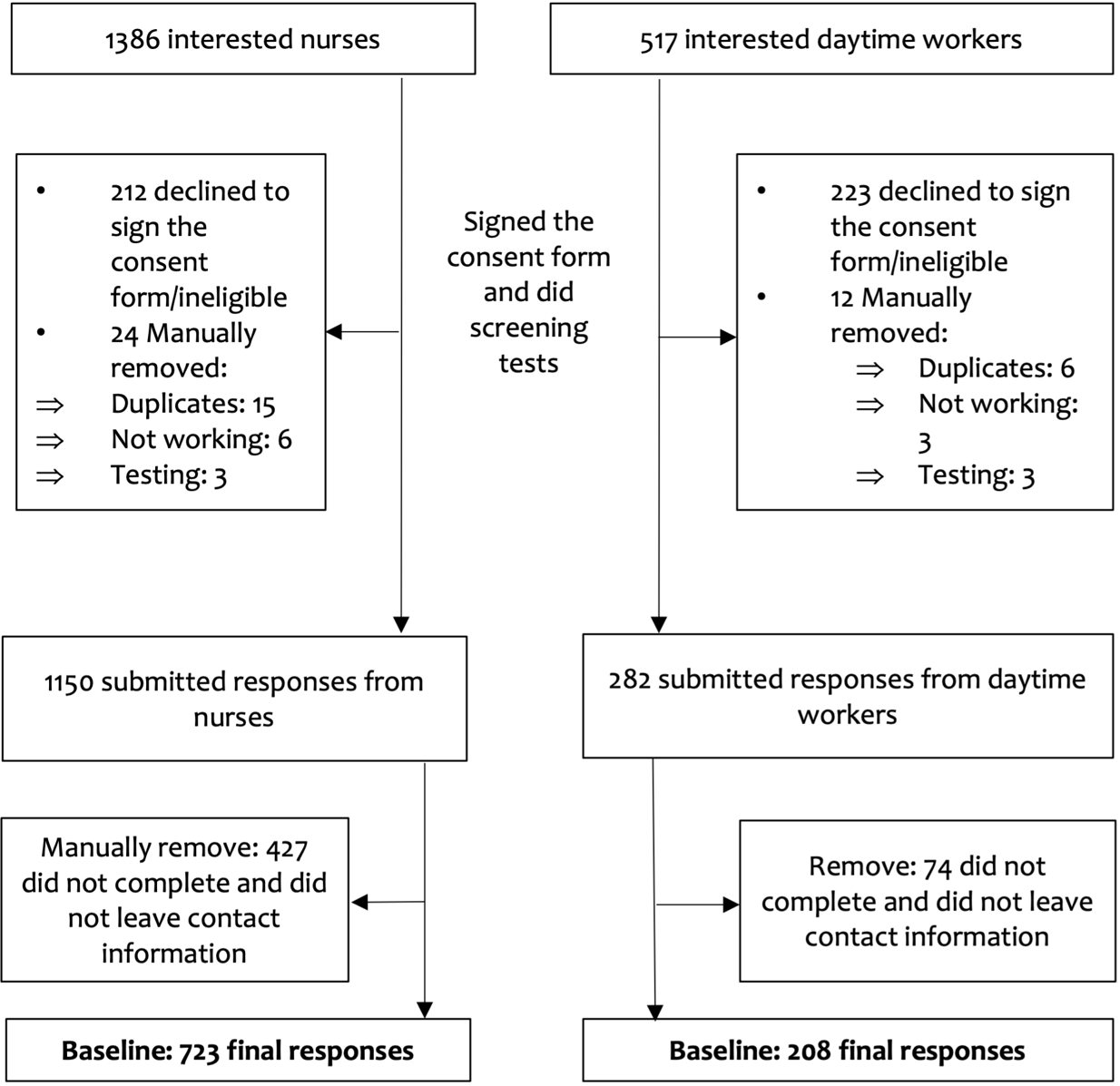

Supplement: Supplementary file 1 — Supplementary Material 1 [file 12912_2025_3990_MOESM1_ESM.docx]
